# A comprehensive regulatory and industry review of modeling and simulation practices in oncology clinical drug development

**DOI:** 10.1007/s10928-023-09850-2

**Published:** 2023-03-04

**Authors:** Ana Ruiz-Garcia, Paul Baverel, Dean Bottino, Michael Dolton, Yan Feng, Ignacio González-García, Jaeyeon Kim, Seth Robey, Indrajeet Singh, David Turner, Shu-Pei Wu, Donghua Yin, Di Zhou, Hao Zhu, Peter Bonate

**Affiliations:** 1 Modeling and Simulation, Metrum Research Group, Tariffville, CT USA; 2 Present Address: Clinical Pharmacology and Pharmacometrics, Gilead Sciences, Foster City, CA USA; 3 Clinical Pharmacology, F. Hoffmann-La Roche AG, Basel, Switzerland; 4 Present Address: Clinical Pharmacology and Clinical Biomarker, Molecular Partners, Schlieren, Switzerland; 5 Oncology Modeling and Simulation, Takeda, Newton, MA USA; 6 Clinical Pharmacology, Genentech, South San Francisco, CA USA; 7 Clinical Pharmacology, Genmab, Copenhagen, Denmark; 8 Simulations Plus, Cognigen, Alicante, Spain; 9 Early Development Analytics, Novartis Institutes for BioMedical Research, Cambridge, MA USA; 10 Quantitative Pharmacology and Pharmacometrics, Merck, Rahway, NJ USA; 11 Clinical Pharmacology, Gilead Sciences, Foster City, CA USA; 12 Clinical Pharmacology and Pharmacometrics, BioNTech SE, Boston, MA USA; 13 Clinical Pharmacology, Early Oncology, Pfizer Inc., New York, NY USA; 14 Clinical Pharmacology, Modeling & Simulation, Amgen Inc., Thousand Oaks, CA USA; 15 Division of Pharmacometrics, Office of Clinical Pharmacology at FDA, Silver Spring, MD USA; 16 Clinical Pharmacology and Exploratory Development, Astellas, Northbrook, IL USA

**Keywords:** Oncology, Exposure–response, C-QT, Markov, Semi-mechanistic, Disease progression, Logistic regression, Time-to-event, Tumor growth dynamics

## Abstract

Exposure–response (E–R) analyses are an integral component in the development of oncology products. Characterizing the relationship between drug exposure metrics and response allows the sponsor to use modeling and simulation to address both internal and external drug development questions (e.g., optimal dose, frequency of administration, dose adjustments for special populations). This white paper is the output of an industry-government collaboration among scientists with broad experience in E–R modeling as part of regulatory submissions. The goal of this white paper is to provide guidance on what the preferred methods for E–R analysis in oncology clinical drug development are and what metrics of exposure should be considered.

## Introduction

Exposure–response (E–R) analyses of oncology drugs are an integral component in their clinical development [1, 2], both for internal decision making and externally to support regulatory approval. Understanding the relationship between dose, exposure, and response allows the sponsor to demonstrate that they understand the pharmacokinetic-pharmacodynamic (PKPD) behavior of their drug. Variability in pharmacokinetics and pharmacodynamics is well known and there are certain patient subgroups that may be at increased risk for adverse events, e.g., patients with impaired renal and/or hepatic function, or decreased efficacy, e.g., ultrarapid CYP2D6 metabolizers [3]. E–R analysis allows one to predict and/or confirm response in these patient subgroups and whether dose modification is needed in these subgroups.

The term ‘exposure’ is a broad one that tries to capture how much drug a person is “exposed” to. Exposure may encompass any or all of the following:

- Some aspect related to dose, such as the daily dose or total cumulative dose a patient receives;
- Some measure of drug concentration in the body at some point in time, such as maximal concentration (C_max_), average concentration (C_avg_) or trough concentration at steady-state (C_trough_);
- Time above some threshold, such as time above some minimum effective concentration, or
- May include integrated measures of concentration, for instance area under the curve at steady-state (AUC_ss_), cumulative area under the curve, or dose at the time of some response.

Indeed, there are many different possible choices for quantifying exposure over time and space (e.g., systemic or tumor). Similarly, ‘response’ is also a broad term that may encompass any or all of the following:

- Assessment of drug efficacy: response rate, time to progression, progression free survival (PFS) or overall survival (OS);
- Assessment of drug safety: whether a patient experiences nausea or vomiting, or the severity of a rash a patient may develop during treatment; or
- Measurement of pharmacodynamic biomarkers: degree of phosphorylation of some important protein, or level of receptor occupancy, etc.

Sponsors may submit several E–R analyses in support of a regulatory submission that encompass different measures of exposure and different measures of clinical outcomes. Identifying dose/dosing regimens that provide a favorable benefit/risk profile is critical in drug development [4–6]. Sponsors may also conduct E–R analyses for internal decision making for go/no go decisions or to inform dose selection for Phase 2/3 trials.

In modern oncology, the old paradigm that “more is better” with the maximum tolerated dose (MTD) being used as the dose studied in late phase studies is no longer the norm. The MTD is commonly defined as the highest dose that most patients can tolerate without unacceptable side effects. However, the introduction of targeted therapies, biologics in general, and immuno-oncology is moving drug development away from the MTD concept in search of the optimal dose/dosing regimen. Recently, the Food and Drug Administration has issued a draft guidance that will require to sponsors to study a range of doses in clinical development with the goal to use the optimal dose in registration trials [7]. As such, alternative approaches to clinical development will be required for dose optimization, facilitating the use of E–R analysis by including safety and efficacy information from more than one dose level.

One of the earliest, most comprehensive, E–R analyses was presented by Houk et al. [8] in patients with advanced solid tumors, including patients with gastrointestinal stromal tumor (GIST) and metastatic renal cell carcinoma (mRCC) treated with sunitinib. Their analyses used different measures of exposure: dose, drug systemic concentration, AUC_ss_, and cumulative AUC during 1 cycle of treatment. Using a combination of population PKPD modeling, repeated-measures logistic regression, and correlation analyses, authors demonstrated that increased sunitinib exposure was associated with longer time to progression, longer overall survival, and a greater chance of clinical response. They also showed that increased exposure was associated with increased blood pressure, increased incidence of fatigue, and a greater probability of neutropenia. These analyses were supportive of the recommended dosing regimen in GIST and mRCC patients that was approved at 50 mg once daily for 4 weeks every 6 weeks (4 weeks on/2 weeks off). Recently, a less intense dose regimen has been proposed for sunitinib: 50 mg for 14 days every 3 weeks (traditional “2/1 schedule”). Both regimens have the same dose intensity (4 weeks on) and both have a 2-week drug holiday period every 6 weeks. The model from Houk et al. mathematically explains why the alternate schedule (2/1) presents less toxicity, and therefore, it is better tolerated [9].

Typically, due to lack of established standard methods, there is no one standard approach to conducting an exposure–response analysis. For example, when performing time-to-event analysis, Kaplan–Meier (KM) curves are a useful graphic assessment for the exploration of observed data. Further analysis such as Cox proportional hazards regression model, parametric time-to-event models, or an accelerated failure time model could also be considered based on the nature of the data. All of these are equally valid and have different assumptions with different pros and cons, and different prediction outputs. Furthermore, it is important to mention that E–R analyses in oncology using survival metrics, may be subject to selection bias and immortal time bias [10]. Landmark analyses and multi-state analyses have been proposed to correct the inherent selection bias resulting in part from the fact that responders must live long enough for response to be observed [11–13]. Therefore, the question arises as to which method should be used and if there is a preferred method of choice. In an attempt to address these concerns, this white paper is the output of an industry- government collaboration between scientists with broad experience in E–R modeling in oncology. The goal of this white paper is to provide guidance on the preferred methods for E–R analysis commonly used in oncology and the measures of exposure to consider in different scenarios. To make things easier for the reader, an executive summary of recommendations and comments from each of the following sections is presented in Table 1.

**Table 1:** Executive summary

Topic	Recommendations and comments
Exposure metric considerations	• This is a key component of any analysis and may include dose, concentration, time-averaged concentration, time above a threshold, or area-related metrics • Be careful of dose adjustments and dropouts, and their effect on exposure metrics • In choosing a metric consider whether the relationship may be a direct effect (like nausea/vomiting) or a time delay effect like tumor growth
Safety and efficacy endpoints: categorical endpoints	
• Examples: presence/absence of nausea/vomiting, presence/absence of grade 3 or higher neutropenia, RECIST	• Primarily logistic modeling (or some modification thereof) is used • 10 Events/predictive variable is recommended for precise estimation of regression coefficients • Consider the confounding effect of drug clearance on the outcome (higher clearance may lead to poorer treatment outcomes); use clearance as a covariate in the model • If time-dependent categorical endpoints are of interest, use Markov models
Safety endpoints	
• Time course of myelosuppression	• Empirical approaches (e.g., maximum % decrease from baseline vs. AUC) o Empirical models have limited values extrapolating outside the doses or dosing regimen tested o Need to use some integrated measure of exposure like AUC because of time delay between first dose and peak effect • Semi-mechanistic models, like a Friberg model, allow assessment of time course of myelosuppression
• QTc interval prolongation	• Recommend following Garnett white paper [63] • Oncology trials may not be able to study 2x-above therapeutic exposure for safety reasons • 20 ms is generally accepted as the upper safety threshold compared to 10 ms in healthy volunteers
Efficacy endpoints	
• Time to event: survival	• Can use nonparametric (Kaplan–Meier), semiparametric (Cox proportional hazard), or parametric (accelerated lifetime) models • KM curves often assessed by quartile of exposure vs OS or PFS • CPH models should include other prognostic covariates, like baseline tumor size and drug clearance, for controlling these confounding effects • May be subject to inherent selection bias and immortal time effects
• Tumor growth dynamics	• Allows for a better understanding of the entirety of a patient’s tumor burden growth/shrinkage time-course to assess the possible impact of dose or schedule selection on disease response • Many different models to choose from • Secondary parameters may be more intuitively linked with survival outcomes in time-to-event analyses • Informative censoring may affect parameter estimates • Pretreatment tumor growth trajectories may allow better interpretability • Rely on prespecified target lesions which may not be indicative of overall disease burden
Hematologic malignancies	
	• All recommendations in the above sections can be applied to hematologic malignancies, where total target tumor size is replaced by the appropriate continuous tumor burden metric for that particular malignancy • May require bounded endpoint models, e.g., minimal residual disease
Tumor biomarker and disease progression	
	• Example: PSA kinetics or circulating tumor cells • May require semi- or mechanistic models to explain
Immunogenicity	
	• May affect both exposure and response (safety/efficacy) • Need to consider anti-drug antibodies (ADAs) vs neutralizing antibodies • Covariate analysis may include binary grouping (presence or absence of ADAs) or titer in models
Cell therapies	
	• CAR-T cells display strange kinetics compared to traditional small molecules or biologics • 4 Phases including distribution, expansion, contraction, and persistence • Cannot use allometric principles for scaling of doses

Each topic in the table is covered in the text. Bulleted recommendations and comments are excerpts from the text. Read text for further details

## Exposure metrics considerations

When performing E–R analyses, one of the first decision points in the analysis is the exposure metric of choice. One needs to consider what type of data are available, the duration of treatment, and dose compliance. It is not uncommon in oncology for patients to experience dose modifications during the course of treatment [14, 15]. Often, these dose adjustments happen because of adverse events (AEs) that require either a dose holiday or dose reduction. The result of the dose adjustments could lead to lower average drug exposure for subjects with long treatment duration. Further, a high percentage of dropouts is expected due to AEs associated with concurrent exposure. Thus, under this situation, an E–R analysis for some of the most common efficacy endpoints (overall survival, OS, or progression-free survival, PFS) may suggest an inverse relationship between exposure and efficacy. To eliminate the bias introduced by the large percentage of dose reductions, an earlier exposure metric prior to any dose modification (Cycle 1) could be considered. However, this early exposure metric will have limited value in establishing any relationship between exposure and response. Under scenarios where AEs are leading to significant dose adjustments, it is important to consider if the right dose has been identified and whether there is any longitudinal model for efficacy endpoints or surrogate endpoints of efficacy that can be evaluated to better understand the E–R relationship. The oncology dose-finding workshop organized by FDA and the American Association for Cancer Research (AACR) in 2016 presented levantinib as an example of dose adjustment integrated E–R analysis (DAIER). For levantinib, FDA suggested an E–R analysis using dose-altering AEs models to evaluate different dosing regimens and efficacy [16].

Typically in E–R analyses, drug exposure is assumed to be the cause, and response to be the outcome. However, if disease progression or remission influences pharmacokinetic (PK) parameters over time, this interaction between treatment response and PK parameters could result in artificial E–R relationships. Anti-programmed death-1 (anti-PD1) immunotherapies nivolumab and pembrolizumab exhibited time-dependent pharmacokinetics and a correlation between drug clearance changes over time and survival rates [17–19]. In these situations, directly linking drug exposure at steady state to clinical outcomes in a single-dose trial may yield an over-steep E–R relationship, deviating from the true underlying relationship. Interestingly, the nivolumab baseline clearance had a strong association with survival, relative to all evaluated exposure and covariates in a multivariable E–R analysis [20, 21]. Wang et al. showed that baseline nivolumab clearance can be predicted by a composite of cytokine signatures using machine learning approach and the patients with predicted high nivolumab’s clearance (CL) is associated with poor survival regardless of treatment (nivolumab or chemotherapy) in patients with advanced melanoma and renal cell carcinoma (RCC) [22, 23]. These results support the hypothesis that nivolumab CL can be used as a prognostic marker for patient disease status. Moreover, this highlighted the importance of including more than one dose level in E–R analysis to reduce the confounding effect between exposure and CL. It has been demonstrated by Liu, et al., through simulation, that using exposure variables observed or derived from the first treatment cycle for an E–R analysis may minimize this bias [18]. Furthermore, confounded relationships between baseline risk factors for survival and drug exposure have also been reported, complicating the choice of exposure metric to use in these circumstances and the interpretation of any observed E–R relationship [20, 24]. An example of confounded baseline risk factors has been reported for trastuzumab, which is indicated for the treatment of HER2 overexpressing breast cancer, metastatic gastric, and gastroesophageal junction adenocarcinoma [25]. Yan et al. performed an exploratory analysis with simulated trough concentration in the first treatment cycle and overall survival. The Kaplan–Meier curves stratified by different exposure quartiles suggested a E–R trend based on the exposure metric. However, the unbalanced distribution of baseline disease burden across different exposure quartiles was responsible of the apparent E–R relationship. To minimize the confounding effect, a propensity matching strategy for adjusting measured confounders, which are defined by a stepwise Cox regression model, was applied. After appropriate matching, patients in the first exposure quartile of trastuzumab show no survival benefit over control [26].

Although dose could be used as the exposure metric of choice, systemic exposure (i.e., plasma/serum/blood drug concentration) often is a more precise metric as it accounts for nonlinearities and inter-individual variability in the pharmacokinetics of the drug. Depending on the type of E–R analyses, metrics of early exposure (and its correlations with surrogates of efficacy), exposure at steady state, or the time course of drug concentration (time-varying concentration) could be used.

Another important consideration when choosing the exposure metric is potential dose regimen comparisons. Prediction of response in other schedules of administration based on just one dose schedule often will lead to inaccurate outcomes. However, when information is available from more than one dose schedule, evaluating the most sensitive metric of exposure (e.g., C_trough_, C_max_, C_avg_) for clinical outcomes may help to appropriately account for differences in dose schedules. In dose-escalating studies, looking at concentration or dose versus biomarker changes as surrogates of efficacy or proof of biological response could help dose selection and establish the maximum tolerable dose that will lead to maximum biological response. Further, it is worth mentioning that in order to collect sufficient information over an informative range of doses or exposure, an adaptive/Bayesian design could be a good choice. However, such study designs may cause logistical and operational challenges [27].

Therefore, the use of the drug exposure metrics depends on the study design, the drug mechanism of action, and the nature of the relationship between exposure and response (i.e., short versus long-term effects). In oncology when there is a single read on efficacy (i.e., objective response rate, ORR), it may be more appropriate to use a simple metric that represents the drug exposure over the course of the treatment. In this case, C_avg_ or C_trough_ could be good metrics of choice since these provide an average measure of exposure; however for E–R safety analyses with acute AEs, the C_max_ prior to the AE event could be explored. Cumulative AUC is confounded with time on study and careful consideration should be given to the use of this metric, as well as the nature of the endpoint under study. AUC at steady state (AUC_ss_) is a valid exposure metric often associated with long-term effects. However, when looking at steady state metrics (ie., C_trough_, C_avg_, AUC_ss_, C_max,ss_) for a given dose schedule, those may be correlated and selecting one metric versus another will often not lead to different conclusions.

Another consideration is the use of model-predicted drug exposure versus observed concentration values. In the case of a drug with very high variability in exposure (i.e., > 70% residual error) and sparse PK sampling, model-predicted exposure profiles may be questionable and the use of observed C_trough_ values over the course of the treatment could be a more reliable exposure metric.

In summary, a variety of exposure metrics could be considered when performing E–R analysis; what should guide the selected drug metrics for E–R is multifactorial and includes the type of E–R analysis, endpoint under consideration, understanding of the drug pharmacokinetics and mechanism of action, the available drug exposure data, and the nature of safety and efficacy endpoints under analysis. Table 2 provides a summary of exposure metrics used for different E–R analyses and clinical response (efficacy/safety) endpoints.

**Table 2:** Summary of types of analyses and exposure metrics to consider

Exposure metric	Analysis	Endpoints
Single exposure metric representing the course of treatment or exposure to the time of the event: *C_trough_, *C_avg_, *AUC_ss_, *C_max_, C_obs_, *time above a treshold concentration	KM Curves, Cox proportional hazards Logistic regression Empirical myelosuppression models	PFS, EFS, OS ORR, CR, MRD, AE grades, Myelosuppression grades
Longitudinal exposure (time-varying exposure): *C(t), *C_trough_(t), *C_ave_(t)	Multi-state models Parametric time-to-event Markov models Tumor growth dynamics Time-course of myelosuppression Disease progression models	SLD-TTE PFS, EFS, OS, AE events SLD neutropenia, lymphopenia, anemia, and thrombocytopenia, Time-course of biomarkers as surrogates of efficacy

*AE* adverse event, *AUC*_*ss*_ area under the curve at steady-state, *CR* complete response, *C*_*avg*_ average concentration calculated as the ratio of cumulative AUC over the timeframe for the cumulative exposure, *C*_*max*_ maximum concentration, *C*_*obs*_ observed concentration, *C*_*trough*_ concentration prior to dose, *EFS* event free survival, *KM* Kaplan–Meier, *MRD* minimal residual disease, *ORR* objective response rate, *OS* overall survival, *PFS* progression free survival, *SLD* sum of the longest diameter, *TTE* time-to-event

*Model predicted/estimated exposure metrics

## Safety and efficacy endpoints: categorical scoring systems

### Logistic regression

In addition to continuous clinical endpoints, categorical or ordered categorical (ordinal) endpoints, such as graded AEs or ORR, are often considered. Depending on the granularity of data collected and the objective of the analysis, logistic regressions and Markov chain models can be used to analyze these endpoints.

Logistic regression modeling is a widely used approach in E–R analysis that enables characterization of the relationship between drug exposure and ordered categorical clinical outcomes [20, 28–31]. In logistic regression analysis, a linear predictor with a link function is used. The linear regression yields a nonlinear relationship commonly through a logit predictor, but other links could be used, such as probit or complementary log–log models. Logistic regression models the probability of an outcome (binomial dependent variable) based on independent variables such as demographic factors, drug exposure, etc. This type of analysis informs about the likelihood of an event happening.

The characterization of relationships between exposure and clinical outcomes of both efficacy [21] (i.e., objective response, based on short-term tumor response) and safety [32] (i.e., AEs of clinical interest) provides a quantitative assessment of the benefit/risk profile and is often used for dose selection in late-stage (i.e., Phase 3) oncology drug development. In some situations, these results can be utilized for dose recommendation in regulatory submissions (i.e., a rolling submission), if the treatment fits an unmet medical need and short-term clinical outcome (e.g., OR) is promising [33, 34].

The development of a logistic regression model generally includes 3 steps; (1) development of a base model, with an evaluation of the appropriate functional form of exposure metrics that may include interaction terms when the drug is given in combination with another drug; (2) development of a full model, including assessment of covariates of interest; (3) and development of the final model after considering the contribution of all potential variables of interest. Even though a final model can be simplified using backward elimination to achieve parsimony, the presence or absence of parameters of minimal impact in the probability will not result in model prediction differences [35].

A minimum of 10 events per predictive variable analyzed in the logistic regression model is recommended for accurate and precise estimation of the regression coefficients. Peduzzi et al. evaluated the effect of the number of events per estimated parameters in logistic regression. They concluded that less than 10 events per predictive variable can lead to major biases, questioning the validity of the logistic regression analysis if those conditions are not fulfilled [35, 36]. Likewise, the number of covariates to be included in the full model should consider the total events in the analysis dataset to avoid over-parameterization. The utilization of the full model provides the benefit of avoiding biased parameter estimation by accounting for all measured covariate effects. Moreover, the full model avoids confounding among covariates and exposure metrics [20, 31]. A recent E–R efficacy analysis of nivolumab showed how baseline CL was a significant predictor of efficacy endpoints, OR, and OS. Subjects with higher CL had poor efficacy outcomes (i.e., higher risk of death and lower OR). Prior E–R analyses found only apparent E–R relationships that were misleading by ignoring the incorporation of baseline CL. It is important to note that baseline CL was not included as an exposure parameter but as a surrogate of disease status and other baseline confounders. In fact, exposure did not influence the outcome. Subjects within a given dose level with the lowest CL were more likely to be responders. No relationship was found comparing exposure levels and clinical response across the range of doses evaluated. The recent approval of flat dosing of nivolumab (240 mg Q2W, and 480 mg Q4W) away from originally approved body weight normalized dosing (3 mg/kg Q2W) was based on a flat E–R relationship that included both baseline CL and systemic exposure in the model, and that increasing drug exposure was unlikely to improve efficacy outcomes (thus, indicative of maximal response). This example highlights the importance of utilizing a full model approach for trials with limited dose-finding information to assess covariates' effect and exposure levels on relevant clinical outcomes [30, 37].

### Longitudinal logistic regression models with markov elements

E–R analysis for categorical endpoints commonly focuses on correlating the highest AE grades observed during the course of treatment within a patient’s measure of exposure. However, this approach ignores the time course of drug exposure and AE development, and, as such, loses valuable information. Markov models are useful when the outcomes are categorical measures that are monitored continuously over time.

Markov modeling has been widely used in ordered and non-ordered categorical analyses in many therapeutic areas of drug development [28, 38–41]. The Markov models considered in this review are first-order models, Markov chains of second or higher orders are models in which the probability of one event changing depends on 2 or more preceding ones and will not be discussed here. A central concept in Markov models is the transition probability, which models the probability of one event changing to another event (or staying the same). For example, for the 2-event state, like whether a patient develops a rash after starting treatment with a drug, there are 4 transition probabilities. If ‘0’ is no rash and ‘1’ is rash, then the transition probabilities are P_00_, P_01_, P_10_, P_11_ where the subscript refers to a change from the current state *i* to the future state *j*. Compared to logistic regression models where there are no transition probabilities and characterization focuses on the probability of having an event at given drug exposure, Markov modeling provides a description of longitudinal clinical data over time and assumes that (i.) the distribution of future states depends only on the current state and (ii.) is not a function of the whole history of events (first-order Markov models). Given this defined Markov property [42, 43], Markov modeling can capture the onset of events, their duration, and severity as they change over time; it also allows the assessment of an exposure effect on the transition probability from one event state to another state and the severity of the event.

For E–R analyses using Markov models where longitudinal categorical data are modeled, drug exposure over time would be a more appropriate exposure metric than a single exposure measurement, such as C_max_ or AUC up to the event. Time-varying exposure would be more associated with the onset and duration of AEs when the dose was modified or discontinued due to adverse events. The effect of co-medication on transition probabilities can also be considered if co-medications were applied for the treatment of specific AEs and with the resolution of AEs. If a delay between exposure and clinical outcome was observed, effective concentrations derived from an effect compartment can be evaluated. Prognostic factors (e.g., biomarkers) could be included to assess their effects on the severity of an AE during model development.

**Fig. 1:**
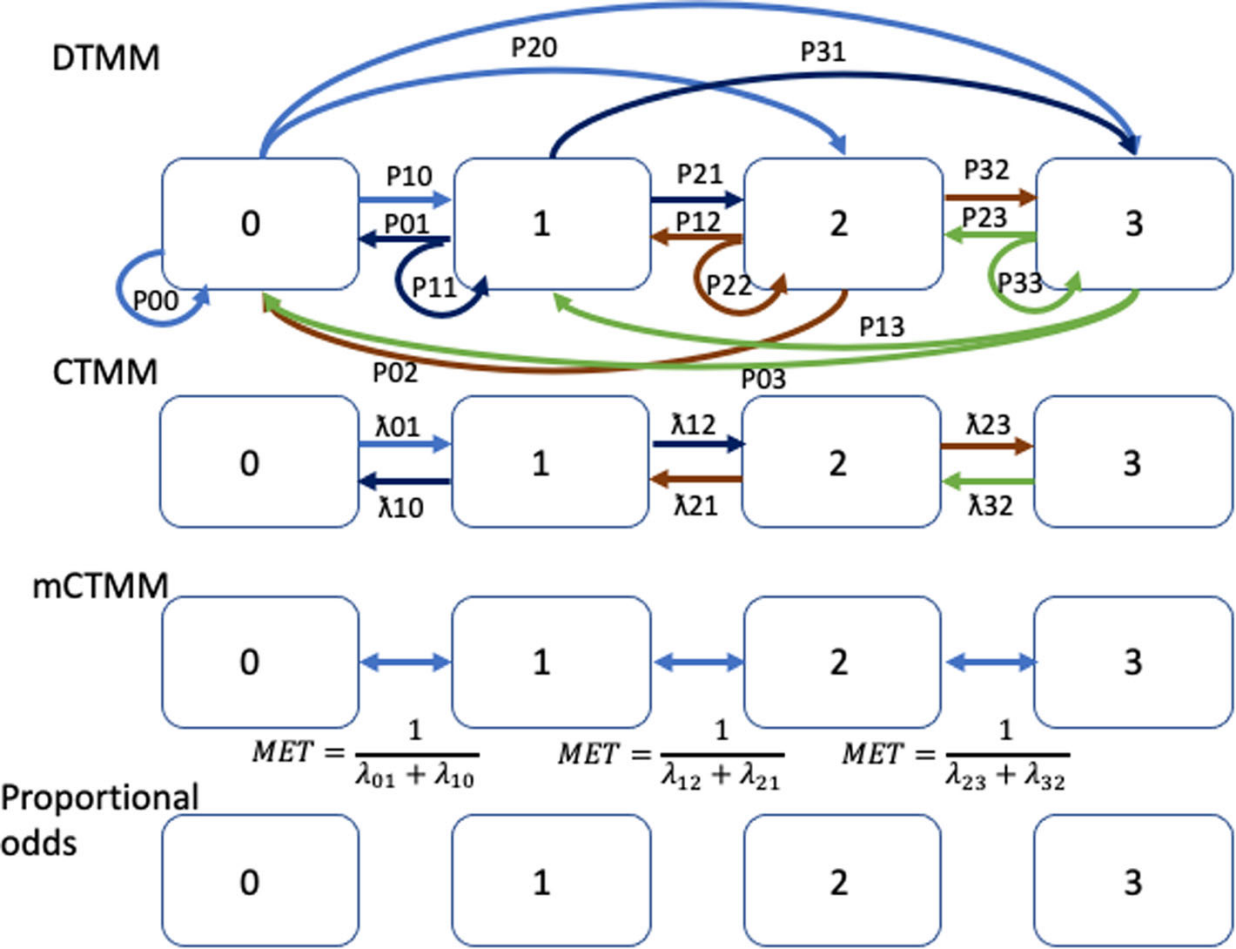
Schematic representation of different Markov models and proportional odds model adapted from Schindler et al. [44]. The preference of selection of one type of Markov model vs. others depends upon the frequency of data collection (time interval of events) and a number of categories (states) of the events. DTMM might be preferred when data were collected with uniform time intervals, whereas CTMM might be suitable for data collected with non-uniform time intervals across patients either due to study design or missing observations. CTMM models, in general, have fewer parameters relative to DTMM, as CTMM assumes that the transitions only occur between neighboring states. mCTMM is a simpler version of the CTMM model, in which mean equilibration time (MET) between two succeeding states is assumed to be constant across states

Different types of Markov models have been reported and will be briefly presented here. The discrete-time Markov model (DTMM) combines proportional odds with a transition model that allows event changes more than 1 grade higher or lower. In this model, all possible transit probabilities can be estimated, with the assumption that the transit probabilities are independent of whatever the time interval is between the two assessments, making it an ideal candidate when we have uniform time interval assessments. The continuous-time Markov model (CTMM) combines proportional odds with a transition model that prohibits event changes more than 1 grade higher or lower. For example, a transition directly from State 3 to State 1 is prohibited, whereas the transition from State 1 to State 2 and then from State 2 to State 3 is allowed, and vice-versa. With a CTMM, the influence of the previous state on the probability of the current state changes with time (usually decreasing over time as the time interval between measurements increases). Thus, this model is preferred when the observation intervals are non-uniform across patients either due to study design or missing observations. Lastly, the minimal CTMM (mCTMM), which is a simplification of the CTMM, is characterized by independent transition rates between two consecutive states and governed by a single parameter, the mean equilibration time (MET). A schematic of these models is presented in Fig. 1. Schindler et al. showed a few examples of model performance between DTMM and mCTMM suggesting that mCTMM had the potential in describing the data reasonably well with a more parsimonious model structure relative to the CTMM [44]. In addition, the effect of covariates (e.g., exposure, biomarker) on the probability of each state can be described in a single relationship. Lu et al. compared the model performance between a proportional odds model, CTMM, and DTMM in which models were developed using weekly based time-course of muscle spasm AE data. Model performance showed that the odds model was influenced by time–frequency, DTMM was unable to describe unevenly spaced data, and CTMM seemed to perform well in all evaluated data frequencies (daily, weekly, and unevenly spaced) [45].

## Safety endpoints

### Time-course of myelosuppression

Blood cell production in the bone marrow is a highly prolific process, making it susceptible to the inhibitory effects of anti-cancer agents with anti-proliferative activities or with immuno-modulatory agents directly targeting markers expressed on the surface of hematopoietic stem cell progenitors or mature blood cells. In fact, myelosuppression manifested as decreases in circulating red blood cells, white blood cells, or platelets, is among the most frequent AEs observed for anticancer therapeutics [46, 47]. Quantitative understanding of the drug effects on blood cell production is important for the assessment or prediction of the myelosuppression risk, as well as the optimization of the dose and regimen, to reduce myelosuppression-related AEs.

The dose/E–R relationships for drug-induced myelosuppression have often been analyzed by empirical or mechanism-based modeling approaches [48, 49]. Empirical models are usually developed by a theoretical understanding of drug behaviors with very few assumptions of the data [50]. Semi-mechanistic disease models use simplified biological systems to describe the available data falling between empirical models and mechanistic models. Empirical modeling approaches involve either regression-based correlation analyses between a descriptor of the blood cell change (such as maximum % decrease from baseline, often called the nadir, or incidence of particular AEs) and the dose or a particular drug exposure parameter [such as the area under the curve (AUC) and time above a threshold concentration] or by empirically linking the dynamic change in blood cell counts to a time-variant drug exposure parameter through a particular function (e.g., E_max_ models). Semi-mechanistic and mechanism-based modeling approaches utilize differential equations to describe the physiological process of hematopoiesis and the pharmacological perturbation by the inducing agents. In practice, the choice of a specific modeling approach in evaluating drug-induced myelosuppression often depends on the purpose of the analysis and the type of data available.

Empirical myelosuppression models are often expressed as the absolute or relative decrease from baseline at nadir, the maximum percentage of decrease from baseline, the duration below a threshold cell count, the area between a threshold line and the observed cell counts vs time curve, or the incidence of a graded hematological adverse event [51]. Typical exposure parameters such as AUC, time above a threshold concentration, and C_max_ may be explored in the analysis. The relationship between drug exposure and the myelosuppressive effects is modeled without regard to the time course of drug concentrations or blood cell counts. This type of correlation analysis may be used to determine the myelosuppression response or outcome associated with a certain dose/exposure level. This analysis is relatively easy to implement and does not require complete blood cell time course data obtained from extensive sampling. However, this type of modeling analysis has no or limited value in predicting myelosuppression time courses or responses beyond the tested dose range and regimens.

Empirical longitudinal models may also describe the time course of blood cell change following drug administration [52, 53]. Because there is a typical delay in the myelosuppressive effect in relation to systemic drug concentrations, empirical longitudinal modeling assumes a direct drug effect from a drug exposure parameter (e.g., cumulative AUC or C_average_), with or without adding a lag time parameter. This empirical modeling allows prediction of blood cell count time courses under certain conditions; however, given the often lack of physiological meaning for the PD parameters in these models, there are also challenges to extrapolate the models to untested conditions in many cases, such as cross-species translation or predicting the effects for similar compounds.

**Fig. 2:**
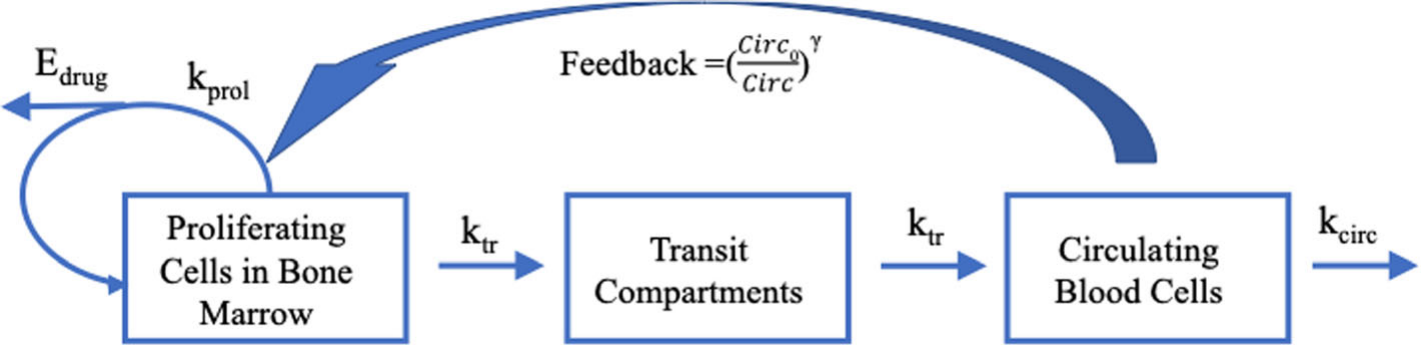
Key structural features of Friberg and related models for myelosuppression. *E*_*drug*_ drug effect, *k*_*prol*_ proliferation rate constant, *k*_*tr*_ maturation rate constant, *k*_*circ*_ circulating cells elimination rate constant, *Circ0* circulating blood cells at baseline, *Circ* amount of circulating cells, *γ* impact factor on feedback

Semi-mechanistic modeling of myelosuppression is based on an understanding of the hematopoiesis process and how the drug perturbs the process. Anti-cancer agents may cause bone marrow suppression through direct cytotoxicity on differentiated bone marrow cells, inhibition of progenitor or precursor cell proliferation, or disruption of growth factor signaling pathways involved in differentiation [54, 55]. There have been generations of mechanism-based mathematical models to describe drug-induced myelosuppression over the last two decades [56, 57]. The most commonly cited model is the Friberg model, which has been the basis of similar models with various modifications [48, 49, 58]. The Friberg model and its related models share the following key structural components (Fig. 2): (1) one or more proliferating compartments with a pool of proliferating cells that can be derived from self-renewable HSCs in the bone marrow; (2) a series of transit compartments representing nonproliferating cells at different maturation stages in the bone marrow; (3) a compartment representing circulating cells with natural turnover; (4) a negative feedback loop where circulating cells regulate the proliferation of bone marrow cells in the proliferating compartment. These structural features are represented by ordinary differential equations, with system-related parameters inherent to the body system and drug-specific parameters that vary by the inducing agents. The drug effects are incorporated into the models in a way consistent with the myelotoxicity mechanisms.

The Friberg and related models have been utilized to describe various types of drug-induced myelosuppression effects including leukopenia, neutropenia, thrombocytopenia, and anemia [51, 59–61]. These models have been able to capture the delay in myelosuppressive effects relative to systemic drug concentrations, and the recovery, rebound and return to baseline for circulating blood cells upon treatment cessation. Despite the complexity of the models, the total number of parameters remains identifiable.

With the mathematical representation of the physiological and pharmacological processes involved in myelosuppression, the models allow the estimation of system-related and drug-specific parameters. The semi-mechanistic basis enhances the confidence in using these models to extrapolate beyond tested conditions. These models have been used to predict the time courses of circulating blood cell profiles from different doses or regimens. Also, the models can be used in the cross-species translation of myelosuppressive effects [62]. Integration of these semi-mechanistic models into population PK/PD modeling can assess the inter-patient variability and influential covariates of drug-induced myelosuppressive effects.

Since the introduction of this semi-mechanistic model, it has become the golden standard approach to model the myelosuppressive effects of chemotherapy. However, due to the lack of clinical data characterizing the upstream processes of granulopoiesis, some assumptions must be made, such as a constant transit time between maturation compartments. In addition, since the semi-mechanistic model is largely data-driven, the predictive ability of this model might be limited compared with the full mechanistic model [63].

Several researchers have extended the applications of this model by incorporating more complex mechanisms and relationships. For example, Quartino et al. integrated granulocyte colony-stimulating factor (G-CSF)—myelosuppression model to describe the dynamics of endogenous G-CSF and absolute neutrophil count (ANC) following chemotherapy [64]. The final model captured both the initial rise in endogenous G-CSF concentrations following chemotherapy-induced neutropenia and the subsequent return to baseline for G-CSF and ANC. This semi-mechanistic model adequately described the time-course of ANC where the feedback mechanism of G-CSF regulated the neutrophil production and maturation in the bone marrow.

### QTc interval prolongation

Since adoption in the ICH E14 guidance in 2015, concentration-QTc (C-QTc) analyses have rapidly replaced Thorough QT (TQT) studies for the assessment of QT prolongation risk during small molecule oncology development [65–68]. However, the complexities of oncology drug development, including the quick pace of development, differences in trial design, co-medications, and risk–benefit profile in the face of life-threatening disease, pose a number of unique challenges in applying this methodology to exclude a risk of QT prolongation or accurately quantifying the effect size when the drug has a known QT liability. The scope of this section relates primarily to small molecule development; as specified by ICH E14, large targeted proteins and monoclonal antibodies have a low likelihood of direction channel interactions, and a thorough QT/QTc study (or C-QTc analysis replacing this study) is generally not necessary unless the potential for proarrhythmic risk is suggested by mechanistic considerations or data from clinical or nonclinical studies [66].

A scientific white paper on concentration-QTc modeling published in 2018 provides clear guidance and recommendations on standardizing C-QT analyses intended for assessing QTc prolongation risk under ICH E14 in healthy volunteers [68]. In addition to guidance on Phase 1 study design in order to support a C-QTc analysis, a pre-specified linear mixed-effects (LME) C-QTc model was proposed, including variations on this model depending on the available data, and provides guidance on exploratory and goodness-of-fit plots for model evaluation [68]. Assumptions of the pre-specified LME C-QTc model, including lack of drug effect on heart rate (HR), adequacy of the HR correction used for the QT interval (i.e., lack of trend on QTcF vs RR plot), lack of hysteresis between concentration and QTc effects, and a linear C-QTc relationship (vs non-linear relationships) should be explored and justified during model development [68].

Several elements that are important components of a C-QTc analysis in non-oncology drug development are typically unavailable for an oncology program—namely concentration and QTc data at a supratherapeutic exposure, and inclusion of data from placebo subjects, as is typically available during single- or multiple-dose escalation cohorts in many non-oncology programs conducted in healthy volunteers [68]. The lack of placebo subjects in oncology trials typically requires that inferences from a C-QTc analysis are drawn based on baseline-corrected QTc (i.e., ΔQTc), rather than baseline-corrected, placebo-corrected QTc (ΔΔQTc). In addition, the lack of placebo data introduces diurnal fluctuation in QTc as a potential confounding factor for drug effect on QTc intervals [69]. The collection of time-matched baseline (i.e., at the same time points to be collected post-dose) in order to account for diurnal fluctuation has been successfully implemented for C-QT analysis of single-arm trials [65, 68, 70]; where only a pre-dose baseline was available, the inclusion of categorical time effects in the C-QTc model has recently been proposed [71]. Where a compound is known to substantially affect HR (i.e., mean change > 10 bpm), the inclusion of a time-matched baseline can allow a patient-specific HR correction to be calculated from baseline QT/RR data, although consensus has not been achieved on the optimal approach [68, 72].

Due to safety concerns as well as the differences in risk–benefit profile in oncology, the highest tested dose and exposure are frequently also the therapeutic exposure, and meeting the requirement for a supratherapeutic exposure is not possible [65, 68]. This may change with recent regulatory requests to greater explore the dose–response relationship in early clinical studies. Currently, however,recent draft revisions to ICH S7B and E14 may enable a greater number of oncology small molecule development programs to meet the supratherapeutic exposure requirement [73, 74]. When a compound meets the definition of a ‘double negative nonclinical assessment’ for QTc prolongation, a supratherapeutic exposure at the ‘high clinical exposure’ (increase in exposure under the effect of intrinsic or extrinsic factors at the maximum therapeutic dose) is required to exclude a positive control, rather than ≥ twofold the high clinical exposure as required under the previous guidance [66, 73, 74]. As discussed in the ICH E14 Q&A guidance documents, in the absence of a positive control or supratherapeutic exposure, there is a reluctance to conclude a lack of effect on QT, however, if the upper bound of the two-sided 90% confidence interval around the estimated maximal effect on ΔQTc is less than 10 ms, the treatment is unlikely to have an actual mean effect as large as 20 ms [66, 74].

The sample size is also an important consideration for C-QTc analysis. While general guidance of 4–8 subjects on drug and 2–4 subjects on placebo across at least 4 dose cohorts has been proposed based on low false-negative and false-positive rates [68], these studies generally included placebo subjects and/or a supratherapeutic exposure typical of a non-oncology program [75–77]. A recent review of QT prolongation risk assessment of small molecule oncology NDAs from 2011 to 2019 found that where a C-QTc analysis was performed, sample size varied greatly, but was generally smaller when the C-QTc dataset was based on data from early phase studies (~ 20–300 patients), and larger where data from later phase studies (or pooled early and late phase) was used (~ 100–800 patients) [65]. It was noted that no clear trend was identified between sample size and the labeling recommendation category to which an NDA was assigned [65].

Other considerations that may be more frequently encountered in oncology include pooling of data from patients with different underlying malignancies across treatment arms or trials in order to increase sample size or dose range, which may increase the risk of confounders such as differences in health status and concomitant medications, as well as in study conduct or ECG acquisition or analysis. When pooling of data is required, between-study differences and potential bias should be evaluated and justified; this may include through exploratory plots and via the inclusion of a study effect variable on key model parameters [68, 78]. Looking at C-QTc relationships with parent compound and/or active metabolites might be required sometimes to characterize QTc effects [79].

## Efficacy endpoints

### Time to event analysis: survival

In analyzing survival data, two functions that are dependent on time are of particular interest: the survival and the hazard function. The survival function S(t) is the probability of surviving at least to time t. The hazard function h(t) is the instantaneous conditional probability of dying at time t having survived to that time. Survival curves can be estimated nonparametrically (i.e., Kaplan–Meier (KM) curves), semi-parametrically (i.e., Cox proportional hazard model), or parametrically (i.e., a lifetime model). The KM method estimates survival curves without the assumption of an underlying probability distribution in the presence of right-tailed censoring. Although easy to compute, it is hard to include covariate analysis, other than by grouping, in the models. Statistical significance between two survival curves can be made using a log-rank test, which tests the null hypothesis that there is no difference between the population survival curves (i.e., the probability of an event occurring at any time point is the same for the populations under comparison) against the alternative hypothesis that they are not the same.

Cox's proportional hazards models are semi-parametric models making fewer assumptions than typical parametric methods. Cox models quantify how specific factors (covariates) influence the rate of a particular event happening at a given point in time. This rate is commonly referred to as the hazard rate. The hazard function can be written as a multiple linear regression of the logarithm of the hazard with the baseline hazard being the intercept term that varies with time:

$$h\left( t \right) = h_{0} \left( t \right) \cdot e^{{\left( {b_{1} \cdot x_{1} + b_{2} \cdot x_{2} + \ldots + b_{n} \cdot x_{n} } \right)}}$$

where t represents the survival time, h(t) is the hazard function determined by a set of covariates where the coefficients (b_1_, b_2_…b_n_) quantify the effect of covariates on the hazard, h_0_ is the baseline hazard. The quantities exp(b_i_) are called hazard ratios (HR). An HR > 1 increases the hazard indicating a positive effect of the covariate with event probability and therefore negatively associated with the length of survival. An HR < 1 reduces the hazard and increases the probability of survival. The main assumption of the Cox model is that the hazard is proportional: if an individual has a risk of death at some initial point that is twice as high as that of another individual, then at later times the risk of death remains twice as high, that is, the hazard ratio comparing two groups is constant over time. However, in oncology, the assumption of proportional hazard implicit into Cox’s models may not represent the ideal choice where the factor changing the hazard may vary with time.

Unlike the Cox regression model which does not specify the distribution function of the hazard, there are several parametric models such as Weibull, Gompertz, exponential, log-normal, and log-logistic models where the hazard function has to be specified. These parametric models allow us to estimate the effects of covariates on the hazard function, including variables that may change over time like drug concentration/dose, age, tumor growth dynamics, or time since surgical intervention.

**Fig. 3:**
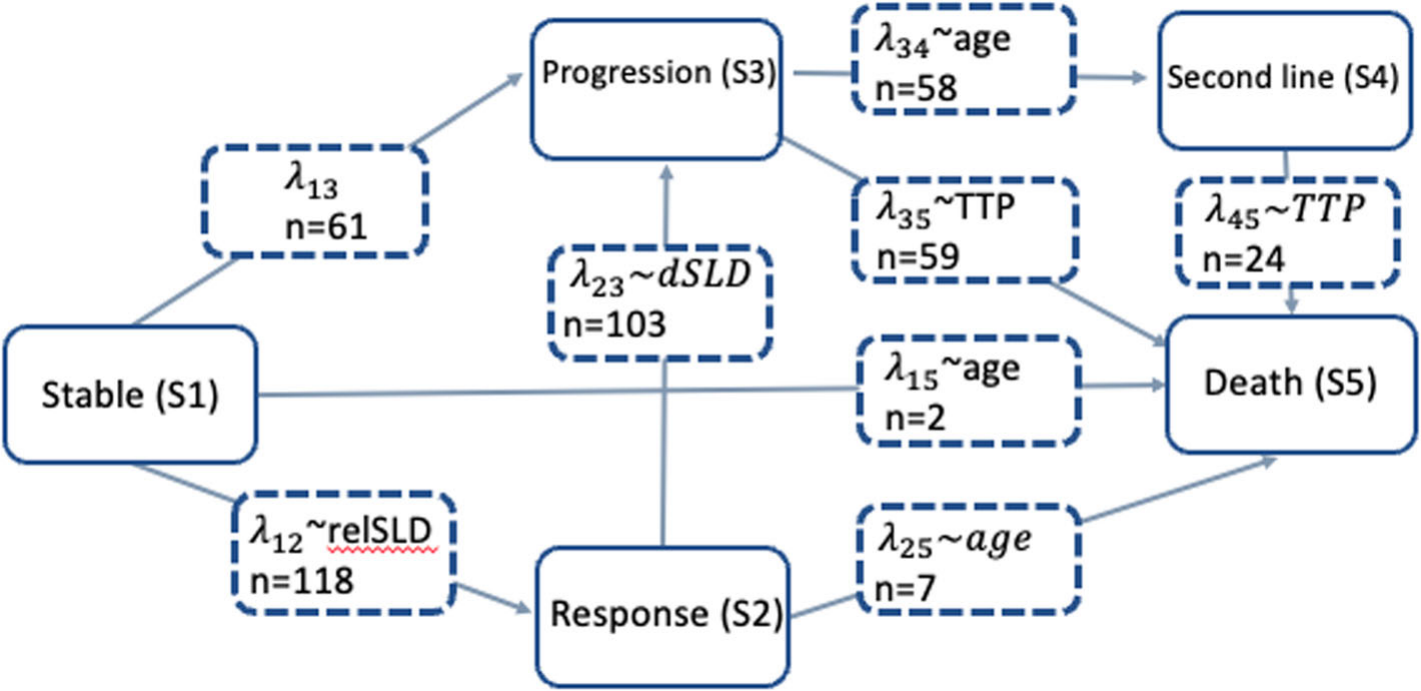
A diagram illustrating the multistate model describing the different states and transitions in the patient population studied. λij represents the transition intensities between states, n for the transitions represents the number of observed transitions, n for the states is the number of clinical outcomes at the end of the study. *dSLD* change in SLD between previous 2 measures, *relSLD* relative change from baseline, *SLD* sum of the largest diameter (tumor data); *TTP* time to progression. Adapted from Krishnan et al. [13]

It is important to highlight the inherent selection bias and immortal time bias in survival metrics in oncology [10]. The TTE model approach with the estimation of a single survival function has its limitations. One alternative approach is the landmark method, which determines each patient’s response at some fixed time point, with survival estimates calculated from that time point and associated statistical tests being conditional on patients’ landmark responses excluding patients that die before the selected landmark timepoint [12]. Another approach to address the challenges of correctly describing the hazard function over time is the use of multistate models. Beyer et al. developed a multistate model with transition hazards estimated using semiparametric models [11]. Krishnan et al. proposed transition hazards using a parametric approach and mixture models [13]. Five states were considered according to Response Evaluation Criteria in Solid Tumors (RECIST) and using predictions derived from a longitudinal tumor growth inhibition model. The model did not allow to move back from one state to another when a response level has been achieved and maybe changed over time. Transition rates between states were estimated and defining the different hazard distributions (see Fig. 3).

In oncology, analysis of the association between drug exposure levels and late-phase clinical outcomes, such as OS and PFS (or event-free survival EFS) are often limited in scope for regulatory submissions. In light of the paucity of data and time constraints, industry practice and regulatory expectations to rationalize the dose rely on performing logistic or Cox proportional hazard regression analysis [20] of the registration-intent trial efficacy data. Even simpler exploratory approaches are sometimes considered to support the dose rationale, such as conducting a KM exploratory analysis of OS and/or PFS stratified by quartiles or tertiles of exposure levels [80, 81]. The general strategy is to demonstrate the absence of a relationship between exposure and the registrable endpoint within the range of exposure tested and infer from this that the dose is optimal since no substantial gain in efficacy can be achieved by increasing exposure levels further. Whether such analyses can be qualified as best practice is contentious as pragmatism is the main driver to rationalize the methodological framework used. Typically, the establishment of a recommended phase II dose is endorsed based on combined analyses of emerging safety signals and surrogate efficacy endpoints with proven independent prognostic value for OS or PFS (e.g., tumor burden [82] or objective response [37, 83, 84] obtained from earlier trial data). Confirmatory trials rarely study more than one dose, and inherently suffer from underpowered statistical consideration for E–R characterization of OS and PFS. Furthermore, the ever-increasing pace for regulatory filing driven by commercial incentives and rapid access of novel treatment to patients in unmet needs further restrains the possibility to develop a data-driven E–R model of OS or PFS for regulatory submission given the time-consuming nature of these analyses. The downside of simple analyses lies in identifying spurious relationships due to imbalance of known prognostic factors of OS at baseline in strata of exposure [85, 86]. For example, ramucirumab E–R showed a positive association with OS in gastric cancer patients following a Cox regression [87, 88] with C_min,ss_ or C_avg_. However, the E–R model did not evaluate covariates such as C-reactive protein levels and tumor burden that are known prognostic factors of OS and could also impact PK as demonstrated in the case of tremelimumab [80], trastuzumab emtansine [81], and to a lesser extent with trastuzumab in the same indication [20]. The recommendation in such a case would be to first interpret with great caution any apparent trend of E–R emerging from simple analyses of trials evaluating a single-dose regimen. To explore further the risk of potential confounding in the event of a positive E–R finding, more sophisticated methods such as the parametric hazard model and multistate models should be attempted to quantify E–R in a multivariate framework. A full model or a selection of covariates of clinical relevance for the endpoint of interest and for the PK metric chosen should then be applied to quantify the relative contribution of covariates on the underlying E–R. As the last step, exposure should be removed from the model in case its causative link with OS or PFS assumed by the model is no longer supported by the data and can be fully explained by prognostic or predictive covariates remaining in the model. The best example of this good practice would be the already mentioned nivolumab E–R analysis in which baseline CL was included in the model [21].

Technical considerations, such as the choice of the PK metrics (C_max_, AUC) or when this metric is measured (first cycle vs steady-state), and data limitations (infrequent PK sampling, usually one dose tested in confirmatory trial) further constitutes challenges to a robust evaluation of E–R. Systemic exposure is consistently used in oncology as the driver of the E–R of OS and PFS while tumor penetration is often non-uniformed across solid tumors. Furthermore, some drugs, such as antibody–drug conjugates (ADC) or combination therapies, require understanding on which exposure level is assumed causally related (e.g., warhead or total antibody exposure) and would require more complex analyses to disentangle the contribution of components. Combination therapy is another challenge from a modeling standpoint since data are generally limited. Monotherapy arms are seldomly available to fully characterize each single agent E–R relationship, and assumptions on the additivity or synergistic nature of the combinatory agents are left without data to infer their validity.

From a regulatory standpoint, simpler E–R approaches are customary to inform label claims justifying the dose as long as several conditions are fulfilled [86]. First, clinical data should demonstrate meaningful benefit-risk for patients for the registrable endpoint and a statistically compelling primary analysis of trial-level data at the given dose, with minimal dose reduction, delays, or omission after treatment initiation. Second, the regulatory strategy implies pre-specifying the model-based analysis plan to reinforce its confirmatory nature. If a 2-stage analysis is selected, individual post-hoc estimates from the popPK model will be incorporated as part of the analysis dataset and use to calculate the metrics of exposure used in the TTE analysis. Alternatively, a simultaneous modeling of PK and response could be carried out with longer running times, more intensive computational power requirements, and in many cases not a clear benefit in final model results unless the response is affecting the drug disposition and the proposed model is accounting for it. Ideally, data from Phase 2 trials with more than one dose level would be ideal to start model development and be prepared to streamline the critical path activities for filing regulatory dossiers. The reality is that due to the life-threating condition of this therapeutic area and the possibility of expedited pathways for approval, phase 2 trials or expansions of Phase 1 trials are often the basis for initial approvals. Therefore, there is usually no possibility of externally validating these analyses with independent (test) data sets. Thus, the analyst is generally not expected to perform external model validation.

Fortunately, more complex models relating OS or PFS with systemic exposure levels, tumor growth dynamics and accounting for dropout, and dose reduction/delays or interruptions are developed once the regulatory submission timeline pressure unwinds [89]. These multivariate tools are far more valuable in their demonstrated track records of impacting the drug development strategy and post-marketing study designs. Leveraging the ability to integrate data from multiple studies and extrapolation/interpolation intrinsic properties [90], these models are used to bridge subcutaneous vs. intravenous dosing, convert flat vs. weight-based or BSA-based [30, 91], adults vs. pediatrics, ethnicity considerations, extend the dose interval [92], redefine therapeutic window for earlier line of therapies, inform patients treatment strategy [93], integrate historical data [94] and other advanced analytic framework [95].

### Tumor growth dynamics

RECIST is the current standard for determining how well a patient’s tumor responds to treatment using assessments of growth/shrinkage captured in on-study X-rays, computerized tomography (CT), or magnetic resonance imaging (MRI) scans. RECIST is broadly accepted by oncology practitioners and regulatory bodies, and nearly all clinical trial treatment assessments for solid malignancies apply this framework. Still, standard RECIST methodology and its criteria for declaring treatment ‘response’ versus ‘non-response’ based on certain % of tumor shrinkage in the original reported tumor lesions, is often critiqued as inadequately representing overall disease burden [96], e.g. at times penalizing a deeper response with shorter time to a progression from nadir [97]. At the center of all RECIST-based assessments, including newer versions such as irRECIST [98], is a practice of categorization of data-rich longitudinal tumor size information into response strata of Progressive Disease (PD), Stable Disease (SD), Partial Response (PR), and Complete Response (CR). These categories are often further dichotomized into binary assignments of responders (PR + CR) versus non-responders (SD + PD) subgroups summarized at the population level as an ORR %. Hematologic malignancy studies use similar categorical response criteria to classify continuous assessments of disease burden, such as percent blasts in Acute Myeloid Leukemia (AML) [99], BCRABL/BCR ratio in Chronic Myeloid Leukemia (CML) [100], and M-protein in multiple myeloma (MM) [101]. In all cases, this categorization leads to loss of statistical power and is insensitive to both time dependencies and depth of response (or non-response) information captured in underlying time-course data. Indeed, if, as in MM, a discretized response spectrum has over six categories including “Very Good Partial Response (VGPR),” it may be a sign that the limits of discretization are being over-stretched to describe an underlying continuum! For this reason, longitudinal tumor burden modeling has become an increasingly applied tool for describing efficacy outcomes in clinical trials and relating tumor dynamics to predictive factors, including treatment dose/exposure [102].

Longitudinal modeling allows for a better understanding of the entirety of a patient’s tumor burden growth/shrinkage time-course to assess the possible impact of dose or schedule selection on disease response. Such analyses are attractive also because they permit derivation of simple secondary parameters to describe features of the profile (e.g. time to re-growth/nadir, depth of nadir, etc.) through interpolation—and sometimes extrapolation—of the observable data. Secondary parameters may be more intuitively linked with survival outcomes in TTE analyses, and thus play an important role in communicating modeling results with a clinical audience. Previously described tumor burden models have been employed in a wide array of drug development applications, and a significant number of these models successfully applied in late-stage development are simple, empirical models with a minimal combination of linear or exponential primary parameters describing growth (e.g., Kg) or shrinkage (e.g., Kd) of target tumor(s). Details on many of these kinetic tumor burden models have been previously published [82, 103–106] including several excellent review articles [107–109].

Central to the notion of longitudinal tumor burden modeling is the incorporation of multiple time-point observations per patient to describe an overall disease trajectory. The type of data incorporated will inherently impact model fidelity/interpretability, and hence, the utility of its application. In oncology, where ethical/logistical considerations dictate the availability of tumor assessments before treatment initiation and after discontinuation, the influence of underlying data structure on tumor model inferences deserves particularly close attention [110]. Often, when a patient’s disease progresses due to tumor burden growth, the patient will discontinue study medication and contribute little or no more data beyond the treatment discontinuation date. Conversely, patients with responding disease tend to remain on study longer, thus contributing more and *longer* duration of scan data. Those patients with responsive disease, therefore, tend to receive more cumulative therapy and are more likely to experience safety-related dose modifications, which are common in oncology clinical trials due to the long-term systemic toxicities of many antineoplastic treatments. All these factors influence E–R interpretations and require careful consideration. The emphasis here, and for any astute longitudinal modeler, should always be on handling selective missingness of data, or informative censoring. In particular, this can problematically impact multiple aspects of E–R tumor burden modeling [110].

**Fig. 4:**
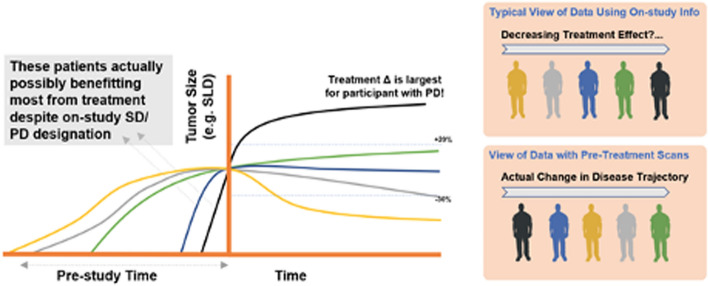
Deeper understanding of treatment effect and exposure–response patterns through tumor burden dynamics modeling: longitudinal modeling allows for robust, quantitative characterization of data-rich pre- and on-study tumor size information on the importance of understanding pre-baseline tumor size trajectory. This hypothetical example illustrates the concept of variability in the tumor growth trajectory at study start and consequent possibility to declare RECIST progressive disease in a patient likely benefiting more from treatment (e.g. black) than another who would be classified with a more favorable stable disease designation (e.g. green). More precise estimates of treatment effect and dose/exposure–response relationships can be brought through a quantitative understanding of a patient’s entire tumor size trajectory, including available pre-baseline scans

Informative censoring often contributes to issues of tumor model parameter identifiability in cases where very little on-study data was collected in one or more patient subgroups. In particular, the time-truncation of data from patients with rapidly progressing disease may limit the availability of data to describe accurate rates of tumor (re-)growth. As such, typical models with simple growth and decay terms to describe tumor kinetic profiles tend to be more empirical than mechanistic in nature, and any biological interpretation of a given parameter with regard to cancer cell replication, treatment resistance, and cell killing is often confounded. In non-linear mixed-effects models, this could manifest as high shrinkage of the estimated between-subject variability of one or more parameters. Parameter variability and parameter estimates could very well be appropriately estimated when the levels of shrinkage are high. However, graphical exploration of covariates using empirical Bayes estimates (EBEs) will not be able to guide covariate search. To mitigate on-study parameter identifiability issues, ideally, one would 1) acquire more data throughout the course of the trial, especially scans following disease progression, or 2) incorporate measurements of pre-treatment tumor growth into the tumor burden model [111] (Fig. 4). Such assessments could be an immensely valuable decision tool as they allow each trial participants’ pre-treatment trajectory to serve as internal control—assuming care is taken in assessing the same set of lesions as the 'future' RECIST target lesions. However, incorporating when “baseline tumor” was collected is relevant information as it is in general days to weeks before the treatment starts. In indications where PFS events are more often related to tumor burden growth (as opposed to survival), this approach then allows projection of control arm PFS using a single (active) arm study [112]. However, due to the nature of most sponsor-initiated studies and focus on the assessment of on-study treatment effects, obtaining and properly digitizing pre-study scans requires additional effort/resourcing and therefore has been rarely implemented.

Another common impact of informative censoring, particularly relevant to E–R applications of tumor burden modeling, involves potentially biased estimates of the E–R relationship when an aggregate exposure summary (i.e. steady-state or cumulative) is applied. A responding individual who remains on the study will necessarily have a higher aggregate/cumulative exposure even if there is no “true” E–R relationship simply because they have had more time on-study to accumulate exposure. A simple best practice in such analyses is to apply an instantaneous, time-varying, or early milestone/baseline exposure metric as the longitudinal model input. Careful consideration of data structure is still required in E–R analyses based on a steady-state exposure/dose intensity which may also be confounded as a result of time-varying dosing due to possible dose reductions and delays. Such analyses could actually imply an inverse exposure–efficacy relationship due to the commonly encountered correlation of time on-study, favorable treatment response, and corresponding increased likelihood of safety-related dose/exposure reductions from the longer duration of exposure. Hence, a general awareness of some of the key aspects of typical oncology trial conduct and these multiple potential confounding phenomena is key in both study design and data analysis to avoid possible misinterpretation of spurious E–R relationships. Similar considerations of informative censoring should also factor into the use of on-study (post-baseline) covariates. Zhen et al. [113] model survival data and longitudinal changes in target lesions accounting for correlation between dropout and response. In this trial with locally advanced or metastatic urothelial carcinoma (UC) patients treated with durvalumab, at the time of the data cut, 100 of 186 subjects had dropped out of the study (67 of them were due to death). As is typical of oncology trials, the risk of patient dropout was strongly influenced by treatment response. Patients with rapid tumor progression dropped out early, whereas those whose disease improved had longer follow-up times. Similarly, Hansson et al. [28] and Schindler et al. [114] incorporated a dropout model enabling prospective simulations of tumor response over time as dropouts were not considered at random.

Tumor burden model structure selection and verification for a particular application should be driven by multiple considerations, including general goodness-of-fit, parsimony considerations, and whether the model is able to adequately describe the data for pre-stated objectives. Prior knowledge on the kinetics of disease burden for a given treatment modality may also factor into model selection. For example, cytotoxic chemotherapy treatments, which differ from immunomodulating treatments in their mechanisms of action, can be expected to show unique kinetics of disease response and progression [115–117]. In general, immunotherapy efficacy has been associated with delayed but durable responses that contrast with the more rapid but transient responses seen with cytotoxic agents. Studies have also shown initial tumor “pseudoprogression” followed by delayed response in some patients treated with immunotherapy [98, 118]. The simpler two or three-parameter empirical tumor models derived primarily from clinical experience with chemotherapy agents often will not adequately capture this type of response pattern. Previous publications describing mixture models have been used to account for and categorize patients with hyper progressive disease as well as those with a delayed, durable response [119, 120].

When choosing a model, careful evaluation of extrapolation bias is recommended given that many existing tumor size models include unbounded exponential growth terms that fail to adequately extrapolate without significant prediction bias [121]. It is therefore critical to examine model performance in the extrapolation setting and to investigate the relationship between follow-up duration and extrapolation bias. Simulation bias tests should generally be performed when evaluating base model structures by first estimating models with time-truncated data, and then assessing the general ability of the model to generate unbiased predictions of ‘future’ data. When assessing model GOF diagnostics, informative censoring and extrapolation bias may also impact interpretation. Visual inspection of trends in conditional weighted residuals versus population predictions, for example, will tend to obscure model misspecification or biased prediction of observations from participants with rapidly growing tumors, which comprise a smaller proportion of the total data set than data from participants with shrinking tumors. VPCs may therefore be a valuable tool for model verification, but again, may also be impacted by extrapolation bias since exponential growth in the post-discontinuation phase can drive anomalous prediction intervals. It is crucial that VPCs also include censoring rules or a drop-out model which approximates clinical practice implemented in the corresponding trial protocols, e.g. truncation of simulated tumor size profiles after 20% growth from nadir—a typical rule governing RECIST progression of target lesions in solid tumor indications.

Advances in radiomics, which applies informatics, machine learning, and other big-data approaches to imaging data, have led to a growth in the number and types of features that may be captured for tumor burden modeling [122]. With appropriate application, this information holds great potential to enhance the clinical relevance of inferences drawn from tumor burden modeling. In alignment with the conventions of RECIST, the majority of published tumor burden modeling studies have been conducted using the summary metric “sum of longest diameters” (SLD) from radiologist-selected target lesions. However, several reports have indicated that volumetric data may be more informative [103]. Hierarchical modeling of individual lesion dynamics with between-tumor variability may yield even deeper insights into disease heterogeneity, as the homogenizing effect of combining lesion information from different anatomic sites for SLD inherently reduces the information available to the modeler.

A key shortcoming of nearly all of the aforementioned tumor burden models, including individual lesion models, is that they rely on information from only pre-specified target lesions—which may or may not be adequately indicative of an overall disease burden. Per RECIST guidelines, individual target lesions are chosen as representative of a patient’s tumor burden for monitoring a relative treatment effect, but a more precise understanding of the tumor size to survival relationships may be established by accounting for the entire tumor/metabolic disease burden [123]. Irrespective of whether unidimensional, bidimensional, or volumetric radiographic assessments are employed, the clinical appropriateness of any given tumor size descriptor should be re-evaluated in different treatment settings and tumor types. While construction of quantitative models linking longitudinal tumor burden with instantaneous survival risk is a relatively recent endeavor, it is already clear that these relationships may vary by nature of different cancer types, anatomical locations of the lesions, and potentially by treatment modality. Hence, the choice of appropriate tumor burden descriptors is likely to be case-dependent and may involve either one or more derived primary or secondary tumor parameters.

### Special considerations in modeling hematologic malignancies

With the exception of lesion-level modeling, all recommendations in the above sections can be applied to hematologic malignancies, where total target tumor size is replaced by the appropriate continuous tumor burden metric for that particular malignancy: M-protein levels in secretory multiple myeloma patients, percent blasts in AML, and BCRABL/ABL ratio in CML. In the latter two, care must be taken to correctly transform the raw disease burden to a bounded assessment value based on the nature of the measurement. For example, in CML, assuming that mRNA levels are proportional to the number of genes in a cell, the BCRABL/ABL ratio can be represented as the ratio of a number of malignant cells to the weighted sum of normal cells (which have two copies of ABL) and malignant cells (which have one copy of ABL) [124].

Modeling lymphoma data may present additional complexities as response assessments are based on both lesion size (sum of products of diameters, SPD) and metabolic activity (FDG-PET avidity) [125]. If raw scans are available, assessment of metabolic tumor volume (MTV) [126], which is the total number of FDG-PET avid voxels in the scanned region of the patient’s body, are preferable to the dichotomized criteria or sum of products of diameters alone, which does not consider whether the nodes in question are actually metabolically inactive (dead).

An additional feature of many hematologic malignancies is the concept of minimal residual disease (MRD) [127], which typically refers to technology with higher sensitivity to low disease burden than the conventional metrics. For example, 6-color flow cytometry in multiple myeloma can detect down to 0.01% levels of myeloma-transformed plasma cells in the bone marrow, as opposed to M-protein levels in peripheral blood which reach the lower limit of detection of 0.1 g/dL at underlying disease burdens ranging from 0.001 to 1%. Six-color MRD, which in the case of MM has been shown to predict incremental survival benefit with every tenfold decrease in MRD [128], are nonetheless dichotomized into MRD positive or negative categories, despite that these definitions may change yearly as more sensitive assays are developed [129]. For this reason and many aforementioned benefits above, we recommend fitting the tumor burden dynamic model to both the conventional continuous metric (e.g. M-protein levels in g/dL) and the continuous MRD metric (e.g. number of cancer cells per ml of sample) simultaneously, which provides greater identifiability particularly when M-protein is below the limit of quantitation (BLQ), which is often the only time MRD is assessed [127].

## Tumor biomarker and disease progression

Disease progression modeling is utilized to describe the time course of disease status and track disease severity over time. These models usually incorporate biomarker data and clinical outcomes to characterize natural disease progression [130].

In prostate cancer (PCa), prostate-specific antigen (PSA) has been recognized as a biomarker for diagnosis, prognosis, and monitoring of disease activity [131]. PCa is usually characterized as either low-risk non-aggressive (indolent) or high-risk aggressive tumors. While indolent PCa is benign prostatic hyperplasia (BPH) in general, aggressive PCa may lead to cancer-specific morbidity [132]. Identification of the most aggressive PCa cases among all patients diagnosed with PCa could help in the selection of the patients who might benefit from radical therapy. de Charry et al. developed a semi-mechanistic model of PSA longitudinal growth to help differentiate aggressive and indolent PCa at diagnosis [133]. The individual preoperative PSA data from patients with PCa and those with benign prostatic hyperplasia were analyzed using a population kinetic approach and a semi-mechanistic nonlinear mixed-effects model [133]. This analysis demonstrated a greater PSA increase rate by cancer cells than by non-cancer cells, while PSA production rate was greater by benign tissue than by malignant tissue. A significant relationship between the PSA production rate by cancer cells and the probability of D’Amico high-risk group was also identified with logistic regression. Moreover, multivariate tests demonstrated that the PSA production rate by cancer cells, Gleason score, and positive surgical margin status were all significant independent predictive factors regarding relapse-free survival (RFS). This semi-mechanistic model provided a possible means to determine whether a patient is likely to have aggressive PCa before surgery.

In addition to PSA, the count of circulating tumor cells (CTCs) has also emerged as a promising surrogate marker in patients with metastatic castration-resistant prostate cancer (mCRPC) [134]. In 2004, the FDA approved the use of the CellSearch® system for detecting CTCs in cancer patients. It is to date the only approved laboratory test for CTCs and is being used to enumerate the number of CTCs of epithelial origin in a 7.5 mL blood sample [135]. Wilbaux et al. developed a semi-mechanistic model to quantify the dynamic relationships between the kinetics of CTC counts and PSA concentrations during treatment in patients with mCRPC [136]. Their joint model incorporated drug effect kinetics for chemotherapy and hormonal therapy through two different K-PD compartments as no drug concentrations data were available. The treatment effects on both PSA and CTCs were assumed to be mediated through a common latent variable that was interpreted as tumor burden. PSA kinetics were described by an indirect response model, while the CTC kinetics in the total body blood was modeled by a cell lifespan model assuming that the rate of cell loss was equal to the rate of cell production delayed by the lifespan. The dynamic change of CTC counts was considered as a random sampling from a negative binomial distribution. By simulating the kinetics of PSA, CTC counts, and the tumor burden, CTC counts turned out to be more sensitive to the variation of the tumor burden. This model was the first to quantify the dynamic links between the kinetics of PSA and CTC counts during treatment in patients with mCRPC. Although limitations exist, this model demonstrated the potential of using CTC counts as a predictor of treatment response or disease progression in patients with mCRPC.

## Characterization of clinical consequences of product immunogenicity

In recent years, biological products, such as monoclonal antibodies and other therapeutic proteins, have been widely used for the treatment of various cancers. Occasionally, the administered biological product may provoke an immune response (known as immunogenicity) in some patients receiving repeated dosing which may lead to excessive cytokine release and/or formation of anti-drug antibodies (ADAs) [137, 138]. Some unwanted adverse events, including anaphylaxis, cytokine release syndrome, infusion reaction, and other non-acute reactions, may occur as the result of the immune response. The cross-reaction between ADAs and their endogenous counterparts may interfere with certain physiological processes, causing additional safety concerns. Furthermore, the immunological reaction may compromise the drug effect. For example, the presence of ADAs usually affects the drug clearance and decreases drug exposure. Additionally, neutralizing ADAs may interfere with the interaction between the therapeutic protein and its target. Therefore, using modeling approaches to quantitatively characterize the clinical consequences of product immunogenicity may provide insights into the risk and benefit profiles in patient subgroups (e.g., ADA positive and negative) and guide the optimal use of the product [139].

Appropriate quantification of the clinical impact of immunogenicity relies on the accurate acquisition of the data. ADAs titers in serum/plasma have been considered as the major biomarker to track patients receiving treatment with a biological product. Time to the first appearance of ADA (i.e., onset), sustained duration, and level of ADA (i.e., duration) may vary among individuals following chronic treatment. Unfortunately, the ADA level cannot be monitored on a continuous basis. The sampling schedule, therefore, becomes a critical component to characterize the potential ADA changes and to link the ADA changes to clinical consequences. A sensitive, specific, and selective bioassay for ADA is another key factor to ensure data quality. In general, ADA is detected by immunoassay using an ADA-antibody, which may interfere with the coexisting therapeutic protein and endogenous substances in biological matrices. An assay’s drug tolerance, which measures the assay sensitivity in the presence of the therapeutic proteins, as compared to typical concentration levels in patients is important to understand the reliability of the detected ADA [140]. The Global Bioanalysis Consortium (GBC) set up an international team to explore the impact of immunogenicity on PK assessments. The result of the work was a white paper where they presented strategies to assess if changes in drug concentration are due to ADA-mediated changes in clearance or instead a consequence of ADA interference with the bioanalytical assay [141]. There is a multitude of factors that could influence the immunogenicity of biologics. These factors could be classified into disease-, patient-, or product-related factors [142–145]. Immunogenicity as a consequence of a biologic therapeutic administration may result in multiple polyclonal antibodies against multiple epitopes circulating in serum. Each ADA species has its own specificity and binding affinity. As a result, these different treatment-emergent ADA responses may have different effects on the PK/PD of the drug therapeutic: a neutralizing effect on activity by interfering with the drug’s ability to bind to its pharmacologic target, a non-neutralizing effect on activity paired with an effect on the PK, or a non-neutralizing effect on activity with an enhanced elimination of the drug therapeutic [145].

The findings on clinical consequences of immunogenicity for a biological product may be misleading if an inappropriate modeling approach is used. Randomized, well-controlled clinical trials are routinely used to characterize the efficacy and safety profiles of a biological product before it gains marketing authorization. An analysis, for instance, may be conducted to compare overall survival in patients with (i.e., ADA positive group) or without ADA (i.e., ADA negative group) detected anytime during the treatment. However, this analysis may lead to misleading outcomes. The time to the first occurrence of ADA and duration for detectable ADA sustained in plasma appears to be random among individuals during the treatment phase. It should be noted that some patients in the ADA-positive group are the patients with the late-occurring ADA formation, who must already survive long enough with continuous treatment. Thus, the true ADA effect on overall survival may be attenuated (i.e., biased), if the overall survival is directly compared between the two groups. Rather, a landmark analysis comparing patients with or without early (e.g., within 4 weeks after the treatment is initiated) detectable ADA may provide a relatively better angle to characterize the potential impact of ADA formation on drug effect, if adequate data are available. The analysis performed for atezolizumab provides a good example. As demonstrated in OAK study in locally advanced and metastatic non-small cell lung cancer patients, 21% of patients were tested positive for ADA by week 4. The analysis suggested that the presence of ADA early after the treatment initiation (i.e., 4 weeks) may significantly affect the overall survival in this patient population [146].

However, careful consideration must be given to any post-randomization variable, including ADA formation. Kong et al. studied the potential impact of ADA formation on long term benefit in a randomized controlled trial with atezolizumab, in which ADA was not observed in the control arm [147]. ADA status was only observable in atezolizumab-treated patients, and variables that are a consequence of the treatment either preclude observation or affect the interpretation of the clinical endpoint of interest. The authors propose a weighted approach for estimating effects based on Weight Placebo Patients (WPP) approach [148] where the post-baseline strata are observed for every subject. The idea behind this is if ADA can be organized in categories, is the treatment benefit clinically meaningful for all categories of ADA? and is the treatment effect similar between certain or all categories of ADA?. Atezolizumab treated ADA-positive patients showed worse OS relative to ADA-negative patients. Furthermore, ADA-positive patients also showed differences in several prognostic baseline variables compared to ADA-negative patients. The authors concluded after correcting the data for the prognostic baseline variables that hazard ratios were similar for ADA-positive and ADA-negative patient populations.

## Modeling in cell therapies

Cell and Gene Therapy (CGT) have now entered a new area of treating diseases. Although scientific efforts have been in progress for over a century, recent advancements in the last decade have significantly shifted the treatment paradigm with several regulatory approvals [149]. As of February 2023, there were 27 CGT products that were approved by FDA [149, 150]. The proven hypothesis, that either alteration of certain cells (Cell therapy) or certain genes into cells (Gene therapy) may lead to treatment, has made CGT very effective in individualized treatment. CGT is formulated differently from traditional large batch production of a small or large molecule (antibody) drug, and in many instances, CGT is designed specifically for individual patients (for example, autologous cell therapy).

Among several CGT modalities, Chimeric Antigen Receptor (CAR) T cell-based therapy has shown clinical benefits in different cancer types [151, 152]. In this treatment, a patient’s T-cells are modified in the laboratory and specific CARs are introduced to the surface of the T-cell in order to target very specific malignant cancer cells. For the purpose of this review, we will focus on current advancements in the clinical pharmacology of autologous CAR-T therapy only.

**Fig. 5:**
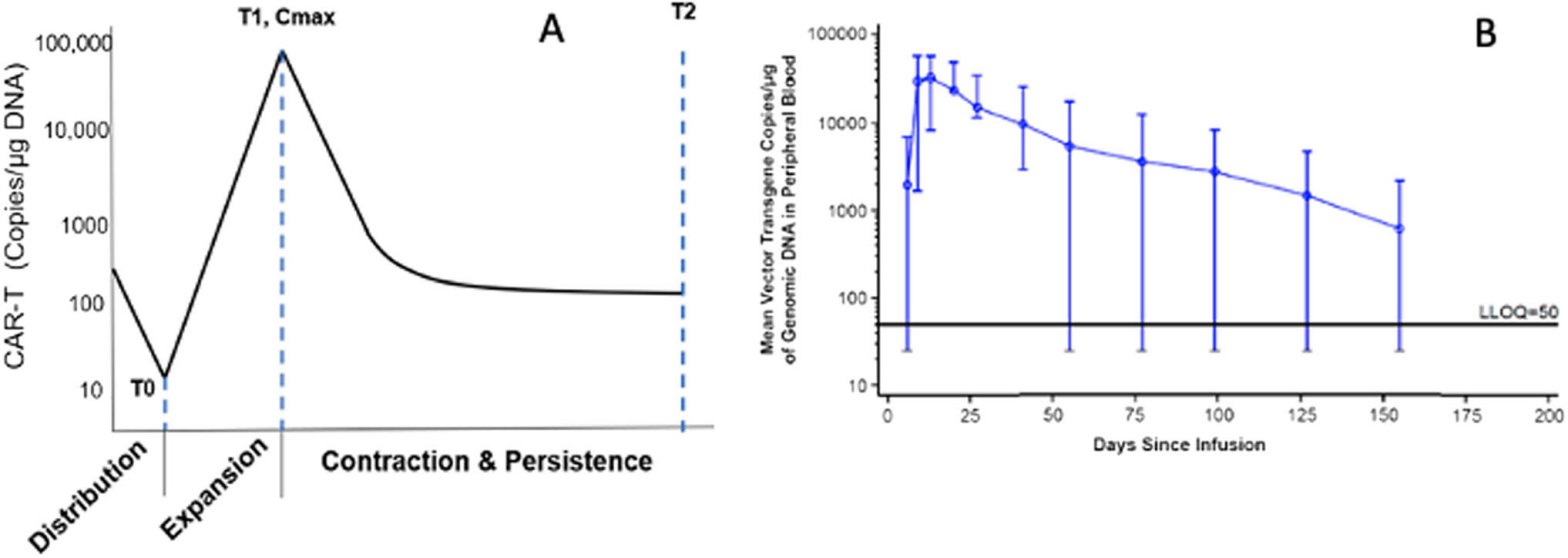
**A** Kinetic Phases of CAR-T therapies. Adapted from Liu et al. [153]. **B** CAR-T expansion and persistence in peripheral blood adapted from Berdeja et al. [154]

The PK of CGT drugs is characterized using quantitative polymerase chain reaction (qPCR) assay for viral products and using flow cytometry or qPCR for autologous cell therapies. A typical small or large molecule drug after IV administration exhibits maximum drug concentration (C_max_) at the end of infusion, whereas for CAR-T cells, the C_max_ is generally achieved after a few days of IV administration due to cellular proliferation and expansion upon interaction with the target antigens in circulation (or at the site of action in tumor or bone marrow). The apparent kinetics of CAR-T may consist of four phases including distribution, expansion, contraction, and persistence [153], see Fig. 5A and B.

In humans, the expansion/proliferation of CAR-T cells is found to be highly variable and primarily driven by an individual's immune system activity. Cilta-cel (Carvykti™) CAR transgene concentrations showed maximum peripheral expansion at a median of 12·7 days (range 8·7–54·6) with observed persistence lasting over > 100 days in peripheral blood [154]. Among patients with 6 months follow-up, most had cilta-cel CAR transgene concentration below the level of quantification (< 50 CAR gene copies per μg DNA) in peripheral blood. Several factors, such as prior lines of therapy, tumor burden, and disease status, may impact an individual's immune system activity. In addition, CAR-T cell drug product characteristics, such as CD4:CD8 ratio, %CAR + T cells, the constitution of effector, and memory cells [155] may also contribute to cellular expansion/proliferation, resulting in highly variable exposure parameters such as C_max_ and area under the curve (AUC) with inter-individual variability as large as 165–190% [156].

Since expansion and persistence of CAR-T cells are dependent on the cellular composition of the individual donor’s original T cells and the individual’s immune system, it is difficult to extrapolate or predict the extent of inter-individual PK exposure (cellular expansion/persistence) in humans. In addition, given the limitation of pre-clinical models in mimicking the complexity of the human immune system, allometric-based principles cannot be applied in translating CAR-T exposure from preclinical species to humans. There have been recent attempts to develop translational models [157], but the lack of relevant preclinical species limits such preclinical to clinical predictions. Current regulatory guidance [158] on gene and cell therapy also states that unlike for small and large molecules, allometric-based scaling cannot be applied to predict starting dose in humans. Typical CAR-T starting dose selection has been based on prior knowledge of cell therapy and target expression.

Since CAR-T exposure exhibits high inter-individual variability, depending on the range of dose levels studied, and the number of subjects at each dose level, dose dependence on PK parameters and impact on response may be difficult to identify. A population cellular kinetic model was developed using clinical data of Kymriah™ [159] where the dose range administered ranged from 0.2 to 5.4 Mcells/kg. The authors did not observe any relationship between dose and exposure. Even though an increase in C_max_ was found to increase the incidence and severity of key safety outcomes (cytokine release syndrome, CRS), due to lack of a clear dose–exposure relationship, such correlations could not be used to identify an optimal dosing regimen. In another study using Abecma® [155], the authors proposed a dose-dependent increase in expansion/persistence based on a prospectively designed Phase 1/2 study where dose escalation was conducted in cohorts at dose levels of 50, 150, 450, and 800 Mcells. Despite a dose-dependent increase in exposure, a plateau in exposure was apparent at 450–800 Mcells. Abecma® transgene levels were positively associated with objective tumor response (partial response or better), where responders achieved ~ 4.6-fold higher C_max_ and ~ 5.6-fold higher AUC_(0–28 days)_ in comparison to non-responders [156]. It is unclear if high inter-individual variability with overlapping exposures and lack of a clear dose–exposure relationship influenced the approved dose range of 300–460 Mcells for Abecma®.

In summary, drug exposure after CAR-T therapy has shown high inter-individual variability with overlapping exposures between dose levels. Although higher exposure may be associated with higher responses, the lack of a clear dose–exposure relationship limits the determination of optimal dose levels. For future trials, a dose-escalation study should be considered in early development with rich PK collection throughout the study to inform better characterization of the dose/exposure–response relationship.

## General considerations

Adequate care needs to be taken in performing E–R analyses using data collected from late-phase oncology clinical trials. The majority of Phase 2 and 3 oncology clinical trials are carried out with one dose level, having minimal E–R information available from the dose-escalation part of the Phase 1 study, often in refractory patients where at the most we can get an idea of E–R for safety endpoints, but we don’t expect any efficacy as the target population is normally not the intended population for the projected filing indication. With only a single dose, the range of exposures is often limited, and may make detection of E–R relationships difficult. FDA has long been pushing for a more thoughtful approach to dosing. With the recent release of a draft guidance requesting sponsors to study a range of doses in early development, this may make E–R modeling more sensitive at detecting such relationships.

Performing a comprehensive pharmacometrics analysis of a drug product at the time of NDA/BLA filing is often challenging but probably the most cost-efficient approach when considering subsequent filings in subsequent indications and special populations. We have seen repeatedly with many therapies that disease burden as characterized by multiple risk factors, such as Eastern Cooperative Oncology Group (ECOG) performance score (PS), percentage of prior surgery, and increased number of metastatic sites, may vary significantly among enrolled patients at baseline and usually changes in the course of the treatment. In late-stage cancer, patients with a high disease burden may be associated with increased clearance for some compounds. Usually, these patients tend to show more aggressive disease progression and shorter survival times. Thus, the disease burden becomes a confounder that affects both drug exposure and survival. All these factors in combination may distort the underlying causal relationship associated with the treatment effect. Inappropriate modeling approaches are more likely to happen when very little information has been collected during early-stage development or no input has been requested from the pharmacometrician, which could lead to an erroneous presentation of the underlying E–R relationship when the pivotal trial reads out.

Often pharmacometricians play “catch-up” by having limited time to perform the retrospective analyses required to comply with regulatory agencies’ recommendations with very little influence on requesting adequately powered dose-finding studies during the drug development program. Usually, the modeling comes at the end, when perhaps the necessary assessments were not collected or incompletely collected, and the information available is insufficient to perform an in-depth analysis of the safety and efficacy endpoints and their correlations with exposure. The pharmacometrics community has the tools, the knowledge, and well-described models summarized in this review that can help characterize E–R in drug development and hence better understand the drug behavior and therapy management. However, tools, knowledge, and models are only half of the work, well-designed trials, and informative data collections are the other half and it is very challenging to do both parts in isolation. A collaborative interaction is required among clinicians, statisticians, pharmacologists, pharmacometricians, and decision-makers to succeed in prospectively defining a clinical development strategy tailored towards the identification of the optimal dose regimen and benefit/risk ratio of a given drug product for the intended-to-treat population of patients.
